# Factors related to the role of programme directors in association with quality in postgraduate medical education – a cross-sectional study

**DOI:** 10.1186/s12909-019-1885-3

**Published:** 2019-12-12

**Authors:** Hanna Wijk, Sari Ponzer, Hans Järnbert-Pettersson, Lars Kihlström, Jonas Nordquist

**Affiliations:** 1 Medical Case Centre, Department of Medicine (Huddinge), Karolinska Institutet, 141 86 Stockholm, Sweden; 2 Department of Clinical Science and Education, Södersjukhuset, Karolinska Institutet, Stockholm, Sweden; 3 Department of Research and Education, Karolinska University Hospital, Stockholm, Sweden

**Keywords:** Residency, Postgraduate medical education, Leadership, Professional development, Quality, Educational leadership, Medical education

## Abstract

**Background:**

Educational leaders have been pointed out as being important for quality of medical education. However, their actual influence on the education can be limited. At the postgraduate level, educational leadership and its connection with quality is underexplored and knowledge about how to increase its impact is lacking. An increased understanding could be used in order to prioritize actions for strengthening the role. The aim of this study was to investigate factors related to the role of programme director associated with quality in postgraduate medical education.

**Methods:**

A cross-sectional study was carried out. A questionnaire was sent to programme directors in Sweden (*n* = 519) comprising questions about background factors, work characteristics, work tasks, hindering and enabling factors, and the Utrecht Work Engagement Scale. A logistic regression and classification tree were used to identify factors associated with high qualitative education, defined as compliance with national regulations.

**Results:**

The response rate was 54% (*n* = 279). In total, 62% of the programme directors reported high quality and factors associated with high quality included experiences of communication with residents, superiors and supervisors, and support from the supervisors. Other factors were consensus regarding postgraduate medical education at the workplace, adequate financial resources, the programme directors’ competence, and their perceived impact on education. Factors of particular importance seemed to differ depending on whether the programme directors were responsible for one or for multiple units. Most high-quality education was found in cases where programme directors were responsible for a single unit and perceived sufficient impact on education.

**Conclusions:**

These results indicated that there was an association between factors related to programme director and quality in postgraduate medical education. The findings pointed out the importance of combining activities at both individual, group and organizational levels. Relational aspects should not be underestimated; faculty development and involvement are crucial.

## Background

A goal for all those involved in postgraduate medical education (PGME) is to contribute to good patient care by providing an education with high quality. Quality in medical education is difficult to define, and thus, there is no consensus on the actual meaning [1, 2]. Quality generally has been defined as, for example, conformance to requirements [3], fitness for use [4], and meeting customers’ expectations [5]. In the context of PGME, quality is often assessed by the fulfilment of different process components or standards [6], in line with the “conformance to requirements” above. The transition from a time- and process-based system to a competency-based framework also influences the view of educational quality [7] and another suitable definition may be “fitness for use” in the sense of producing competent physicians, which can be measured by different assessment methods [8]. In this study, we have defined high quality as the fulfilment of national external regulations. This definition was chosen since it is included in the standards of The World Federation for Medical Education (WFME) [9] and the definition is also used in the Swedish system of quality assurance [10].

The standards of WFME point out the importance of a clearly stated role of professional leadership in PGME [9]. In accordance with this, faculty for PGME usually includes a formal position responsible for specialist education. The conditions, responsibilities, and mandate for this role, here called the programme director (PD), varies [11–13]. In common is the need to handle educational issues in an environment where clinical activities sometimes set conflicting demands and education must be prioritized and negotiated in close collaboration with clinical activities. The role of the PD is often organized as a lower leadership role, a level that is characterized by focus upon co-operative and inspirational leadership and a possible lack of position power [14–16]. The relatively limited impact of leaders in medical education on the educational quality may be a problematic aspect of the role [17–19]. Some educational leaders in PGME are in a managerial position, which implies a greater legitimacy due to their formal leadership role.

Despite of the fact that PDs are identified as being important components of high qualitative PGME and the fact that their impact on education may be limited, knowledge about how to increase their impact is lacking. The aim of this study is to investigate which factors related to how PDs perceive their role are associated with high quality in PGME.

## Methods

### Context of the study

In Sweden, responsibility for providing health care is devolved to the county councils and, in some cases, municipal governments. Each county defines the needs per specialty and finances suitable caregivers to organize and implement physician’s specialist training, which can be offered by most hospital departments and health care centres for primary care. The admission and selection of residents is done locally, which means that there is no national admission. The National Board of Health and Welfare regulates the process of specialist training in Sweden, formulates national educational outcomes for each specialty and approves applications for specialist competence after completion of training [10]. The regulations include a mandatory PD function. The PD’s area of responsibility can relate to one or several training units and the role is most often managed as a part-time duty alongside work as a consultant. Some departments have chosen to organize the role of the PD to include a managerial position vis-à-vis the residents. The total number of PDs in Sweden is unknown, since there is no central registration.

### Participants

This study was carried out in Sweden and is part of a larger project being conducted, with a focus on the role of PD in PGME. The sample consisted of all persons registered in a voluntary list server of PDs provided by the Swedish Medical Association. Inclusion criteria were PDs with more than six months’ experience, a time limit we considered to be appropriate for having experience enough to be able to answer the questions, while not excluding persons having the assignment on an annual basis. PDs not working with specialist training, i.e. PDs at an internship level, were excluded.

### Data collection

The Swedish Medical Association contacted individuals who fulfilled the inclusion and exclusion criteria by e-mail, asking those not wishing to participate, as well as those who were no longer PDs, to contact them. The final sample consisted of 519 individuals. Participants were invited by e-mail during December 2016. In addition to a link to the questionnaire, information was given on the study, outlining the voluntary nature and the extent to which the data would be used and maintained confidentially. Non-responding participants received three reminder e-mails about the survey before the survey was concluded in February 2017.

### Instrument

The questionnaire was based on the results of two studies earlier in the project [11, 20] and on the Utrecht Work Engagement Scale (UWES) [21]. The survey contained 30 questions divided into five distinct sections. First, the survey gathered information about (1) demographic and role-specific factors and high quality. The second section contained (2) questions about the quality of the PGME at the participants’ workplace. Subsequently, the survey gathered information on (3) work tasks. The participants were asked to indicate the extent to which they performed different tasks on a six-point scale. They were also asked to indicate how important they considered the task to be. The next section of the survey (4) sought to measure the experience of enabling and hindering factors influencing the PD’s performance of work tasks. Respondents were asked to indicate their level of agreement with each statement on a 5-point scale. To further validate the responses in this section, the respondents were asked to mention the three most important factors hindering their performance of work tasks. The answers were consistent with the outcome of the qualitative study on which the questionnaires were based (i.e. the obstacles mentioned in the free answers were the same as the alternatives in the questionnaire). Finally, the last section of the instrument was (5) work engagement measured using the UWES [21] in its Swedish version [22]. The questionnaire was pilot tested on 13 persons, leading to minor changes in the final version.

### Variables

The outcome was the quality of PGME, defined as fulfilling the national external regulations. Five paragraphs with mandatory regulations were chosen. These criteria are in accordance with the Accreditation Council for graduate medical education programme requirements [23].

1. All residents have individual training programmes that comply with the requirements in the description of objectives.
2. The individual training programme is regularly followed up and revised if needed, in consultation with the PD, supervisor, and resident.
3. The resident has access to supervisors during each training period.
4. The resident’s development is continuously assessed based on the description of objectives and their individual training programme throughout the whole specialist training.
5. The continuous assessment is made using known and agreed assessment methods.

The participants should indicate to what extent they considered the statements to be consistent with the PGME at their workplace on a four-point scale (1 = totally disagree, 2 = disagree, 3 = agree, 4 = totally agree). The outcome was dichotomized into high or low quality. High quality was defined as those people who assessed their own workplace as three or four on all five questions. Thus, low quality was one or two on at least one or more of the questions.

To study which factors were associated with high quality, the following overarching dimensions were chosen: demographic background, the prerequisites of the role (e.g. type of role, time), work tasks, hindering and enabling factors influencing the performance of work task and engagement (according to UWES). These factors were primarily related to contextual and role-specific aspects of the role which have been described in earlier studies [11, 20, 24]. However, none of the previous studies has examined the relationship between these factors and the quality of education. In a similar way as for the outcome, the work tasks and hindering and enabling factors were dichotomised. For the questions on work tasks, “yes” was defined as those people who answered 5–6 on the six-point scale (5 = a fairly large part, 6 = a large part), whereas “no” was defined as those who answered 1–4 (1 = never, 2 = a small part, 3 = a fairly small part, 4 = neither or). For the questions on enabling and hindering factors, “yes” was defined as those who answered 4–5 on the five-point scale (4 = agree to a high degree, 5 = totally agree), whereas “no” was those who answered 1–3 (1 = totally disagree, 2 = agrees to a low degree, 3 = partly agree).

### Statistical procedure

All analysis was done in the Statistical Package for Social Sciences (SPSS version 23; SPSS Inc., Chicago, IL, USA). Study participant characteristics were analysed using descriptive statistics, i.e. frequencies, percentages, and mean values. We used logistic regression to study the relationship between high quality and the factors chosen.

Our model strategy was as follows: first, we studied the unadjusted association between high quality and each separate factor in univariable models. Second, we used a multivariable model to adjust for the six factors that we considered to be associated with high quality but that were not possible to influence: gender, years in practice, years as PD, medical specialty, the organizational level of the role and number of residents. We then added the other factors, one a time. Thus, the adjusted results could be interpreted as associations with high quality after adjustment for fixed factors. A *p* value of < 0.05 was regarded as statistically significant.

We also used classification tree to identify PDs that confirmed high quality and to identify factors associated with high quality. We used the CHAID algorithm to build the tree [25] including all variables in Tables 1, 2, 3 and 4. A CHAID analysis starts with all the data in one group. Each possible split on each independent variable is considered, in order to find the split that leads to the strongest association with high quality. Tree depth was limited to five levels, minimum parent node was set to 30 and minimum child node was 20. No split with Bonferroni adjustment of less than 0.05 was executed.

**Table 1:** Associations between demographic and role-specific factors and high quality in postgraduate medical education. % = the proportion within the group that answered 1, quality. For example, 183 of the respondents were female. Among these 66% reported high quality

	Number of individuals	% high quality	*P*- value	Crude OR	*P*-value	Adjusted OR^a^
Gender			0.13		0.2	
Female	183	66%		1.5 (0.9–2.5)		1.4 (0.8–2.5)
Male	96	56%		1.0		1.0
Years in practice			0.9		0.7	
< 10	31	61%		1.0 (0.5–2.3)		1.2 (0.7–2.3)
10–19	136	61%		1.0		1.0
> 19	111	64%		1.1 (0.7–1.9)		1.4 (0.6–3.7)
Years as programme director			0.05		0.17	
0.5–2 years	94	53%		1.0		1.0
3–7 years	127	69%		2.0 (1.1–3.5)		1.8 (1.9–3.4)
> 7 years	58	62%		1.4 (0.7–2.8)		1.6 (0.7–3.7)
Medical specialty			< 0.01		0.02	
Auxiliary	34	71%		3.3 (1.3–8.4)		2,8 (1–7.7)
General practitioner	59	51%		1.4 (0.7–3.1)		3.6 (1.4–9.4)
Medicine/neurology	50	42%		1.0		1.0
Paediatric	21	71%		3.5 (1.1–10.4)		3.4 (1–11.4)
Psychiatric	20	75%		4.1 (1.3–13.2)		5.8 (1.7–20.3)
Surgical	75	75%		4.1 (1.9–8.8)		4.6 (2–10.6)
Other	19	63%		2.4 (0.8–7.0)		2.1 (0.7–6.7)
Type of role			< 0.01		0.06	
PD at a single unit	150	72%		2.8 (1.7–4.6)		2.4 (1.2–4.9)
Managerial position at a single unit	17	71%		2.6 (0.9–7.8)		2.0 (0.6–6.9)
PD for several units	112	48%		1.0		1.0
Number of residents			< 0.01		0.04	
0–10	88	69%		2.7 (1.5–5.1)		2.2 (1–5.2)
11–20	73	70%		2.8 (1.5–5.4)		2.3 (1–5.2)
21–30	32	72%		3.1 (1.3–7.4)		3.6 (1.4–9.7)
> 30	86	45%		1.0		1.0

^a^ Adjusted for gender, years in practice, years as PD, medical specialty, type of role, and number of residents

**Table 2:** Associations between work tasks of the programme director and high quality in postgraduate medical education

	Number of individuals	% high quality	*P*- value	Crude OR	*P*-value	Adjusted OR^a^
Organize and plan the process of PGME at the workplace			0.64		0.07	
Yes	209	63%		1.1 (0.7–2)		1.8 (1.0–3.5)
No	70	60%		1.0		1.0
Organize and plan the training of individual residents			0.35		0.6	
Yes	119	66%		1.3 (0.8–2.1)		1.1 (0.7–2.0)
No	160	60%		1.0		1.0
Support and supervise the residents			0.9		0.5	
Yes	107	63%		1.0 (0.6–1.7)		1.2 (0.7–2.1)
No	172	62%		1.0		1.0
Support and supervise the supervisors			0.78		0.25	
Yes	61	64%		1.1 (0.6–2)		1.5 (0.7–3.0)
No	218	62%		1.0		1.0
Handling conflicts and disagreements			0.59		0.6	
Yes	33	67%		1.2 (.6–2.7)		1.3 (0.5–2.9)
No	246	62%		1.0		1.0
Negotiate the residents’ training needs in relation to the need for clinical production			0.33		0.46	
Yes	99	59%		1.0		1.0
No	180	64%		1.3(.8–2.1)		1.2 (0.7–2.2)
Negotiate between different residents			< 0.01		< 0.01	
Yes	42	83%		3.5 (1.5–8.3)		3.3 (1.3–8.4)
No	237	59%		1.0		1.0
Make PGME visible and highly valued			0.26		0.52	
Yes	166	60%		1.0		1.0
No	113	66%		1.3 (0.8–2.2)		1.2 (0.7–2.2)
Own competence development			0.86		0.25	
Yes	78	62%		1.0		1.0
No	201	63%		1.1 (0.6–1.8)		1.4 (0.8–2.7)
Part of the management team			0.40		0.6	
Yes. full or associate	110	59%		1.0		1.0
No	167	64%		1.2 (0.8–2.0)		0.9 (0.5–1.6)
Part of board of research and education			0.28		0.6	
Yes. full or associate	106	58%		1.0		1.0
No	167	64%		1.3 (0.8–2.17)		1.2 (0.7–2.1)

^a^ Adjusted for gender, years in practice, years as PD, medical specialty, type of role, and number of residents

**Table 3:** Associations between hindering and enabling factors and high quality

	Number of individuals	% high quality	*P*- value	Crude OR	*P*-value	Adjusted OR^a^
Sufficient time for the assignment			0.09		0 .24	
Agree	113	57%		1.0		1.0
Do not agree	163	67%		1.5 (0.9–2.5)		1.4 (0.8–2.5)
Clinical activity is such that PDs tasks can be performed as well as possible			0.63		0.22	
Agree	101	64%		1.1 (.7–1.9)		1.4 (0.8–2.6)
Do not agree	171	61%		1.0		1.0
Adequate financial resources			0.07		0.04	
Agree	119	56%		1.0		1.0
Do not agree	141	67%		1.603 (1.0–2.7)		1.8 (1.0–3.3)
Adequate rules and guidelines			0.2		0.45	
Agree	177	65%		1.4 (0.8–2.3)		1.3 (0.7–2.3)
Do not agree	98	57%		1.0		1.0
Education is valued highly enough in the organization			0.29		0.16	
Agree	120	66%		1.305 (0.8–2.1)		1.5 (0.9–2.7)
Do not agree	156	60%		1.0		1.0
Consensus about PGME at the workplace			< 0.01		< 0.01	
Agree	148	72%		2.4 (1.5–4.1)		2.9 (1.7–5.2)
Do not agree	122	52%		1.0		1.0
Sufficient communication with superiors			0.07		< 0.01	
Agree	148	70%		1.6 (1–2.6)		2.2 (1.2–4.0)
Do not agree	128	56%		1.0		1.0
Sufficient communication with residents			0.03		0.03	
Agree	146	68%		1.7 (1–2.8)		1.9 (1.1–3.2)
Do not agree	130	55%		1.0		1.0
Sufficient communication with supervisors			< 0.01		< 0.01	
Agree	74	80%		3.2 (1.7–6.0)		5.0 (2.4–10.4)
Do not agree	198	55%		1.0		1.0
Sufficient support from the supervisors			< 0.01		< 0.01	
Agree	98	76%		2.5 (1.5–4.4)		2.9 (1.6–5.3)
Do not agree	168	55%		1.0		1.0
Sufficient own skills			0.18		0.04	
Agree	186	65%		1.4 (0.8–2.4)		1.9 (1.0–3.5)
Do not agree	89	56%		1.0		1.0
Can influence the education			0.12		0.02	
Agree	104	68%		1.5 (0.9–2–5)		2.1 (1.1–3.9)
Do not agree	173	59%		1.0		1.0

^a^ Adjusted for gender, years in practice, years as PD, medical specialty, type of role, and number of residents

**Table 4:** Associations work engagement and high quality

	Number of individuals	% high quality	P- value	Crude OR	*P*-value	Adjusted OR^a^
Work engagement			0.9		0.19	
Low	41	61%		1.0		1.0
Average	154	62%		1.0 (0.5–2.1)		1.8 (0.8–4.1)
High	84	64%		1.2 (0.5–2.5)		2.3 (0.9–5.8)

^a^ Adjusted for gender, years in practice, years as PD, medical specialty, type of role, and number of residents

### Ethical considerations

The participants were informed in an e-mail about the study’s aims and design, where it was also stated that the results would only be used for research purposes and that participation was voluntary. Ethical approval for this study was applied for from the regional Ethical Review Board (dnr 2012/1662–31/5).

## Results

### Demographic and role-specific factors and high quality

Completed questionnaires were received from 279 PDs representing an overall response rate of 54% (279/519). All Swedish geographic regions were represented as well as all specialty groups, and 66% (183/279) of PDs were female and 56% male (96/279). Their experience as a PD varied between 6 months and 20 years, with an average of 7 years.

In all, 62% (174/279) of the PDs reported high quality on PGME and the percentage varied between different PD roles (Table 1).

The demographic and role-specific factors associated with high quality in the adjusted analysis were medical speciality and number of residents (Table 1).

### Work tasks

The most commonly reported work tasks were to organize and plan the process of PGME at the workplace (75%, 209/279) followed by making PGME visible and highly valued (59%, 166/279). The tasks least reported were handling conflicts and disagreements (12%, 33/279), discussions and negotiation between different residents in the department (15%, 42/279), and to support and supervise the supervisors (22%, 61/279). The work task associated with high quality after adjustment was to discuss and negotiate between different residents (Table 2).

### Enabling and hindering factors

The respondents were asked to mark their level of agreement with different statements connected to enabling and hindering factors. The factors most respondents agreed on were sufficient own skills (reported by 68%, 186/275), and the presence of adequate rules and guidelines (64%, 177/275). The statements that few respondents agreed on were sufficient communication with supervisors (27%, 74/272), sufficient support from supervisors (37%, 98/266), and having sufficient possibility to influence the education (38%, 104/277).

Factors associated with high quality that were felt to hinder or enable the PD in their role were: sufficient communication with residents, sufficient communication with superiors, sufficient communication with supervisors, sufficient support from the supervisors, sufficient opportunities to influence the education, sufficient own skills, consensus about PGME at the workplace, and adequate financial resources. Associations remained after adjustment for factors linked to the PD (Table 3).

### Work engagement

According to UWES, 55% (154/279) reported an average work engagement vis-à-vis their role as a PD, 30% (84/279) high, and 15% (41/279) low work engagement. Quality of PGME was not associated with the PD’s work engagement (Table 4).

**Fig. 1:**
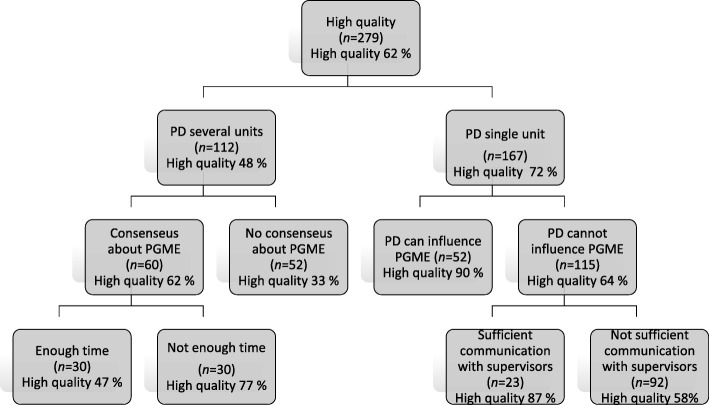
Classification tree showing the factors that at each step had the strongest association with high quality. N = number of PDs in each group. % = the proportion within the group that answered high quality. For example, 52 PDs at a single unit answered that they could influence PGME at the workplace. Among these 90% reported high quality while 10% reported low quality

The classification according to the classification tree analysis showed that the rate of high qualitative PGME ranged between 33 and 90% within different groups (Fig. 1), compared with an overall proportion of high quality of 62%. The PDs with the highest proportion of high quality were PDs in a single unit with sufficient possibilities to influence the PGME (90%, 47/52 high quality). In contrast, the group with the lowest occurrence of high quality (33%, 17/52) consisted of PDs responsible for several units who felt there was no consensus around PGME in the organization. The classification tree analysis also indicated that the most important factor associated with high quality was the type of PD role. For PDs for several units, consensus around PGME was the factor strongest associated with high quality. That is, in workplaces with consensus on what characterized high educational quality, the quality of the PGME was higher. For PDs at a single unit (also including PDs with a managerial position) on the other hand, the experience of being able to influence the postgraduate medical education at the workplace was the most important factor associated to high quality.

## Discussion

The result showed that factors associated with educational high quality included relational aspects (the perceived quality of the communication with residents, superiors and supervisors and support from the supervisors), structural aspects (adequate financial resources, medical speciality and number of residents), individual aspects (the PD’s own skills), and attitudinal aspects (consensus about PGME at the workplace). Also, the PD’s perceptions of their possibility of influencing the education, for example to plan and implement improvements, was associated with high quality, a factor that likely is influenced by the other factors. According to the multi-frame leadership theory of Bolman and Deal’s [26, 27] four essential perspective should be utilised for effective leadership: structural, human resource, political and symbolic. Since PGME is controlled by various formal requirements and policies, and creating formal structures for the training is a responsibility that is often mentioned [11, 12, 23] there is a possible consequence that PDs may focus on the structural perspective. Our findings point to the importance of the human resource perspective of the PD role which therefore focuses on people emphasising support, staff development and communication. Communication is also a tool according to the political perspective, an approach that emphasises negotiation and building power bases when there are competing interests within an organization. The importance of these personal power sources of the PD role is in line with earlier research on power for leaders without formal authority [16], and it has also earlier been argued that informal aspects have greater importance than formal aspects in workplace learning [28].

Clinical supervision is one of the key components of the residents’ learning, which, however, sometimes is lacking in quality [29, 30]. Still, only a minority of the PDs in our study reported that communication with the supervisors was sufficient, nor was support from the supervisors sufficient. Collaboration within the faculty is an important part of the educational leadership in medical education [31, 32]. Based on our results, communication and collaboration between PDs and other key groups within PGME should be given priority in order to create a community of practice [33] around the educational domain. This could be strengthened by educational activities for PDs in combination with creating practical communication opportunities by prioritizing time and creating routines for how and when communication and collaboration will take place.

Many of the factors affecting clinical learning is culturally embedded [28]. A high qualitative PGME requires a framework in which education is not seen as a burden but as having a production value. Our results suggest that a workplace culture which supports educational activities by prioritizing financial and time resources, and has a clear consensus on how that education should be delivered, will strengthen the role of the director and their ability to contribute to high quality education.

This study has some limitations. Firstly, the sample was based on PDs in a voluntary database and the response rate was moderate. As there is no central registration of PDs in the Swedish context, the use of the voluntary database was the only opportunity of reaching the target group. As a result of being voluntary, the database used was not updated; many people only have a PD assignment for a shorter period, and then remain on the list, which may be an explanation. If this dropout resulted in a selection bias that might have affected the result is unknown. However, by controlling the spread of respondents in terms of geographical location, medical specialties, and other background variables, it was found that the respondents had a distribution likely to be representative of the whole population of PDs.

Secondly, this study was limited to PDs’ subjective perceptions. It could be argued that alternative sources of information might have offered a more objective view. However, in this study the use of programme PDs’ perceptions was inevitable because we wanted to explore the association between how the role is experienced by PDs and the educational quality, which involves internal processes and, as a consequence, cannot be assessed objectively. Concerning the validity of the measure of educational quality, the ideal measurement should be outcome of patient care, which obviously is associated with many challenges. As described in the introduction, we used the “conformance to requirements” definition, which means that quality has been operationalized to fulfil a number of formal requirements. This was considered to be most appropriate to be able to replicate the study in other countries. One weakness in the quality measure is the interpretable formulation of the regulations on which the questions about quality rely on (e.g “The resident’s development should be continuously assessed ( …)” ) which could make the inter-rater reliability low. The variation for an individual is probably lower, indicating a high test-retest reliability.

Finally, educational leadership in PGME differs around the world, both in terms of educational culture and formal structures. The generalisability of the study outside Sweden depends on how the PD role is organized, and the result should be read with the contextual background in consideration. Thus, the external validity outside Sweden is unknown and should be investigated further.

## Conclusion

These results indicate that there was an association between factors related to the PD role and quality in postgraduate medical education. The findings pointed out the importance of combining activities at both individual, group and organizational level in order to strengthen the PDs’s positive impact on the educational quality. Relational aspects should not be underestimated and the role and faculty development and involvement are crucial.

We make three recommendations to enhance the quality of education:

1. Create forums that enable communication, collaboration, and peer-to-peer learning within the faculty.
2. PDs should participate in educational activities designed to develop leadership and communication skills.
3. Development of the PD role must be done in parallel with the organization prioritizing educational activities at a more general level.

## Data Availability

The datasets used during the current study are available from the corresponding author on reasonable request.

## Abbreviations

PD: Programme director
PGME: Postgraduate medical education
UWES: Utrecht Work Engagement Scale
WFME: World Federation of Medical Education

## References

[1] Bleakley Alan, Browne Julie, Ellis Kate (2013). Quality in medical education. Understanding Medical Education.

[2] Laura S, Sarah P-E, Heather W, Linda C (2015). Definitions of quality in higher education: A synthesis of the literature. High Learn Res Commun..

[3] Crosby PB (1979). Quality is free : the art of making quality certain.

[4] Juran JM, Godfrey AB (2000). Juran's quality handbook.

[5] Deming WE (1988). Out of the crisis : quality, productivity and competitive position.

[6] Corrigan O, Ellis K, Bleakley A, Brice J, Swanwick T (2010). Quality in medical education. Understanding Medical Education - Evidence, Theory and Practice Wiley-Blackwell.

[7] Ten Cate O (2017). Competency-Based Postgraduate Medical Education: Past, Present and Future. GMS J Med Educ.

[8] Bailey D (2016). Ensuring quality in postgraduate medical education: competency testing is the key. Virchows Arch.

[9] World federation for medical education (2003). WFME Global standards for Quality Improvement. Copenhagen.

[10] Socialstyrelsens föreskrifter och allmänna råd om läkares specialiseringstjänstgöring, SOSFS 2015:8 (2015).

[11] Fryden H, Ponzer S, Heikkila K, Kihlstrom L, Nordquist J (2015). Roles, tasks and educational functions of postgraduate programme directors: a qualitative study. Postgrad Med J.

[12] Malling B, Scherpbier AJ, Ringsted C (2007). What is the role of the consultant responsible for postgraduate education in the clinical department?. Medical teacher.

[13] Lieff S, Albert M (2012). What do we do? Practices and learning strategies of medical education leaders. Med Teach.

[14] DeChurch LA, Hiller NJ, Murase T, Doty D, Salas E (2010). Leadership across levels: levels of leaders and their levels of impact. Leadersh Q.

[15] Bass BM, Bass R (2008). The Bass handbook of leadership : theory, research and managerial applications.

[16] Yukl G, Falbe CM (1991). Importance of different power sources in downward and lateral relations. J Appl Psychol.

[17] Sundberg K, Josephson A, Reeves S, Nordquist J (2017). Power and resistance: leading change in medical education. Stud High Educ.

[18] Malling B, Mortensen LS, Scherpbier AJ, Ringsted C (2010). Educational climate seems unrelated to leadership skills of clinical consultants responsible of postgraduate medical education in clinical departments. BMC Med Educ.

[19] Sundberg K, Josephson A, Reeves S, Nordquist J (2017). May I see your ID, please? An explorative study of the professional identity of undergraduate medical education leaders. BMC Med Educ.

[20] Wijk H, Ponzer S, Heikkila K, Kihlstrom L, Nordquist J. Factors influencing effectiveness in postgraduate medical education – a qualitative study of experiences of the responsible clinical consultants. BMC Med Educ. 2019;19:3. 10.1186/s12909-018-1433-6. 10.1186/s12909-018-1433-6 PMC6318888 30606174

[21] Schaufeli W, Salanova M, González-romá V, Bakker A (2002). The measurement of engagement and burnout: a two sample confirmatory factor analytic approach. J Happiness Stud.

[22] Hallberg UE, Schaufeli WB (2006). “Same same” but different? Can work engagement be discriminated from job involvement and organizational commitment?. Eur Psychol.

[23] Common Program Requirements; effective July 1 , 2017. Accreditation Council for Graduate Medical Education Web site. https://www.acgme.org/Portals/0/PFAssets/ProgramRequirements/CPRs_2017-07-01.pdf. Accessed 22 Nov 2018.

[24] Long TR, Brown MJ, Elliott BA, Rose SH (2010). Characteristics of anesthesiology residency program directors. J Clin Anesth.

[25] Kass G (1980). An exploratory technique for investigating large quantities of categorical data. Appl Stat.

[26] Bolman LG, Deal TE (1991). Leadership and management effectiveness - a mulitframe, multisector analysis. Hum Resour Manag.

[27] Bolman LG, Deal TE (2013). Reframing organizations : artistry, choice, and leadership.

[28] Kilty C, Wiese A, Bergin C, Flood P, Fu N, Horgan M (2017). A national stakeholder consensus study of challenges and priorities for clinical learning environments in postgraduate medical education. BMC Med Educ.

[29] Kilminster S, Cottrell D, Grant J, Jolly B (2007). AMEE guide no. 27: effective educational and clinical supervision. Med Teach.

[30] Kilminster SM, Jolly BC (2000). Effective supervision in clinical practice settings: a literature review. Med Educ.

[31] Lieff SJ, Zaretsky A, Bandiera G, Imrie K, Spadafora S, Glover TS (2016). What do I do? Developing a competency inventory for postgraduate (residency) program directors. Med Teach.

[32] Bing-You RG, Holmboe E, Varaklis K, Linder J (2017). Is it time for Entrustable professional activities for residency program directors?. Acad Med.

[33] Wenger E (1998). Communities of practice : learning, meaning, and identity.

